# Vaginal microbiota and genitourinary syndrome of menopause in premenopausal breast cancer patients receiving endocrine therapy: a longitudinal cohort study protocol

**DOI:** 10.3389/fmed.2026.1826064

**Published:** 2026-06-15

**Authors:** Fangfang Chen, Zhiyuan Bo, Yangfan Fan, Yizhou Huang, Yiding Chen, Fang Wan

**Affiliations:** 1 Department of Surgery, Women’s Hospital School of Medicine Zhejiang University, Hangzhou, China; 2 Department of Gynecology, Women’s Hospital School of Medicine Zhejiang University, Hangzhou, China; 3 Department of Breast Surgery, The Second Affiliated Hospital Zhejiang University School of Medicine, Hangzhou, China

**Keywords:** endocrine therapy, genitourinary syndrome of menopause, premenopausal breast cancer, prospective cohort study, vaginal microbiome

## Abstract

**Background:**

Endocrine therapy with ovarian function suppression combined with aromatase inhibitors or tamoxifen is essential for hormone receptor-positive breast cancer in premenopausal women, but often induces genitourinary syndrome of menopause (GSM) via hypoestrogenism. Existing research focuses on natural menopause, with limited longitudinal data on vaginal microbiome changes in breast cancer survivors, especially amid rising cases among young Chinese women.

**Objectives:**

To (1) assess temporal effects of endocrine therapy on vaginal microbiome *α*- and *β*-diversity and dominant taxa (e.g., Lactobacillus); (2) explore dose–response links between microbiome shifts and GSM severity; and (3) compare OFS + AI versus OFS + TAM impacts on vaginal microecology.

**Methods:**

This single-center prospective cohort at Women’s Hospital, Zhejiang University School of Medicine, will enroll 110 premenopausal women (18–45 years) with hormone receptor-positive breast cancer, allocated to OFS + AI or OFS + TAM based on clinical regimens. Vaginal swabs collected at baseline and 1, 3, 6, 12 months undergo 16S rRNA sequencing (V3-V4 region) with DADA2-based ASV analysis via QIIME 2 for species-level resolution. Assessments include Vaginal Health Index (VHI), GSM symptoms (VAS for dryness, dyspareunia, burning), s, and urinary symptoms (ICIQ-SF). Primary outcomes: microbiome diversity (Simpson index) and Lactobacillus abundance. Secondary: VHI, VAS, urinary scores. Analyses use repeated measures ANOVA and generalized estimating equations; sample size accounts for medium effect (*d* = 0.8) and 10% attrition. Ethically approved and registered in Chinese Clinical Trial Registry.

**Conclusion:**

As a longitudinal study on vaginal microecology in this population, this protocol integrates molecular and clinical data to reveal GSM mechanisms, informing personalized interventions for better quality of life and adherence.

**Clinical trial registration:**

http://www.chictr.org.cn/showproj.html?proj=296570, Identifier (No. ChiCTR2500115283)

## Introduction

Breast cancer continues to be the most prevalent malignancy among women worldwide, with its incidence rate persistently increasing and exhibiting a notable shift toward younger age groups in recent years. According to the most recent global cancer burden data, China reported 357,200 newly diagnosed breast cancer cases in 2022, with 20–30% of these cases occurring in women under the age of 50, and 7% diagnosed before the age of 40 (1, 2). For premenopausal patients with hormone receptor-positive breast cancer, endocrine therapy has emerged as a crucial strategy for enhancing survival outcomes (3). Substantial evidence from randomized controlled trials has demonstrated that ovarian function suppression in combination with either aromatase inhibitors or tamoxifen significantly improves disease-free survival compared to tamoxifen monotherapy (4, 5). Consequently, this combination regimen has been endorsed by authoritative guidelines, including those from the National Comprehensive Cancer Network (NCCN) and the European Society for Medical Oncology (ESMO), as the standard treatment approach for high-risk premenopausal breast cancer patients.

Nevertheless, the majority of young breast cancer survivors undergoing prolonged anti-estrogen therapy, chemotherapy-induced amenorrhea, and premature ovarian insufficiency experience varying degrees of hypoestrogenic symptoms during treatment. Among these, the symptoms associated with GSM are particularly pronounced, including vaginal dryness, burning sensations, dysuria, and dyspareunia, which tend to progressively worsen. Research indicates that approximately 70% of patients develop GSM symptoms, such as vaginal dryness and dyspareunia, following treatment with ovarian function suppression in combination with tamoxifen or aromatase inhibitors (5, 6). These symptoms significantly impair quality of life and subsequently compromise treatment adherence. Research suggests that 20–30% of breast cancer survivors cease endocrine therapy as a result of adverse effects associated with hypoestrogenism (7). In contrast to natural menopause, drug-induced “chemical menopause” is marked by a sudden onset, more severe symptoms, and typically occurs approximately 20 years earlier than physiological menopause, thereby exerting a significant psychological burden on younger women (5, 8).

The vaginal microecosystem is crucial for maintaining the health of the reproductive tract (9, 10). Previous studies have thoroughly elucidates the molecular mechanisms and therapeutic strategies related to the female vaginal microbiome (10, 11). Estrogen deficiency leads to atrophy of the vaginal epithelium and depletion of glycogen, which compromises the survival of Lactobacillus species and results in dysbiosis, increased pH, and exacerbation of GSM symptoms (12, 13). Cross-sectional studies have shown that postmenopausal women exhibit increased vaginal microbial diversity with a significant reduction in Lactobacillus, which strongly correlates with GSM symptoms (14, 15). A multicenter analysis conducted in 2024 demonstrated that non-Lactobacillus-dominant microbial states, such as the enrichment of Gram-negative bacteria, significantly increase the risk of GSM development, further confirming that vaginal dysbiosis is an independent risk factor for GSM (16). Consequently, changes in the vaginal microbiome represent a crucial aspect of the pathophysiology of GSM. The depletion of Lactobacillus species and the proliferation of anaerobic bacteria are not only indicative of the GSM vaginal microenvironment but may also directly induce symptoms via inflammatory and metabolic pathways (15–20). Profiling the vaginal microbiome shows potential as a biomarker for assessing GSM risk, which could inform predictive, preventive, and precision intervention strategies in the management of GSM.

Although the clinical characterization of GSM in perimenopausal and postmenopausal women is well-established, investigations into its relationship with alterations in the vaginal microbiome among breast cancer survivors remain limited. Prasanchit et al. (21) examined 40 postmenopausal breast cancer survivors undergoing aromatase inhibitor therapy. The study found that symptomatic patients exhibited a significantly increased vaginal abundance of Sneathia and Gardnerella, whereas asymptomatic patients showed a higher abundance of Parvimonas. These findings suggest a potential link between alterations in the vaginal microbiome and GSM symptoms. This study was constrained by several limitations, including a small sample size, a cross-sectional design that precludes causal inference, and the lack of serum estrogen validation. A study conducted in Kazakhstan in 2021 examined the vaginal microbiome of 278 breast cancer patients, comprising 174 individuals undergoing combination therapy and 104 former patients. The study identified a significant depletion of Lactobacillus, although no substantial differences in microbial composition were observed across different cancer subtypes. Patients with Luminal A/B subtypes exhibited higher proportions of anaerobic bacteria, whereas those with Her2/Neu + and triple-negative subtypes were associated with specific pathogenic bacteria, such as Staphylococcus (22). However, this study did not investigate the relationship between the microbiome and breast cancer treatment or GSM.

In summary, while recent pioneering studies have begun to explore the longitudinal changes in the vaginal microecology of breast cancer patients undergoing endocrine therapy (23, 24), key knowledge gaps persist regarding the comparative effects of OFS + AI versus OFS + TAM on vaginal microbiota dynamics and the dose–response relationships between microbiome shifts and GSM severity. Therefore, this prospective cohort study aims to further contribute to this growing body of evidence by evaluating the effect of endocrine therapy on vaginal microbiome and GSM. This prospective cohort study aims to: (1) characterize the temporal changes in vaginal microbiome *α*-diversity (Simpson index), *β*-diversity, and relative abundance of predominant taxa (particularly Lactobacillus species) over 12 months in patients receiving endocrine therapy with ovarian function suppression; (2) compare the impact of two common regimens-ovarian function suppression combined with aromatase inhibitors (OFS + AI) versus ovarian function suppression combined with tamoxifen (OFS + TAM)-on vaginal microbiome composition; and (3) explore the associations between these microbiome changes and the severity of GSM symptoms, including potential dose–response relationships, as assessed by validated clinical measures (Vaginal Health Index, visual analog scales, and urinary symptom scores).

## Methods and analysis

### Study design

**Figure 1:**
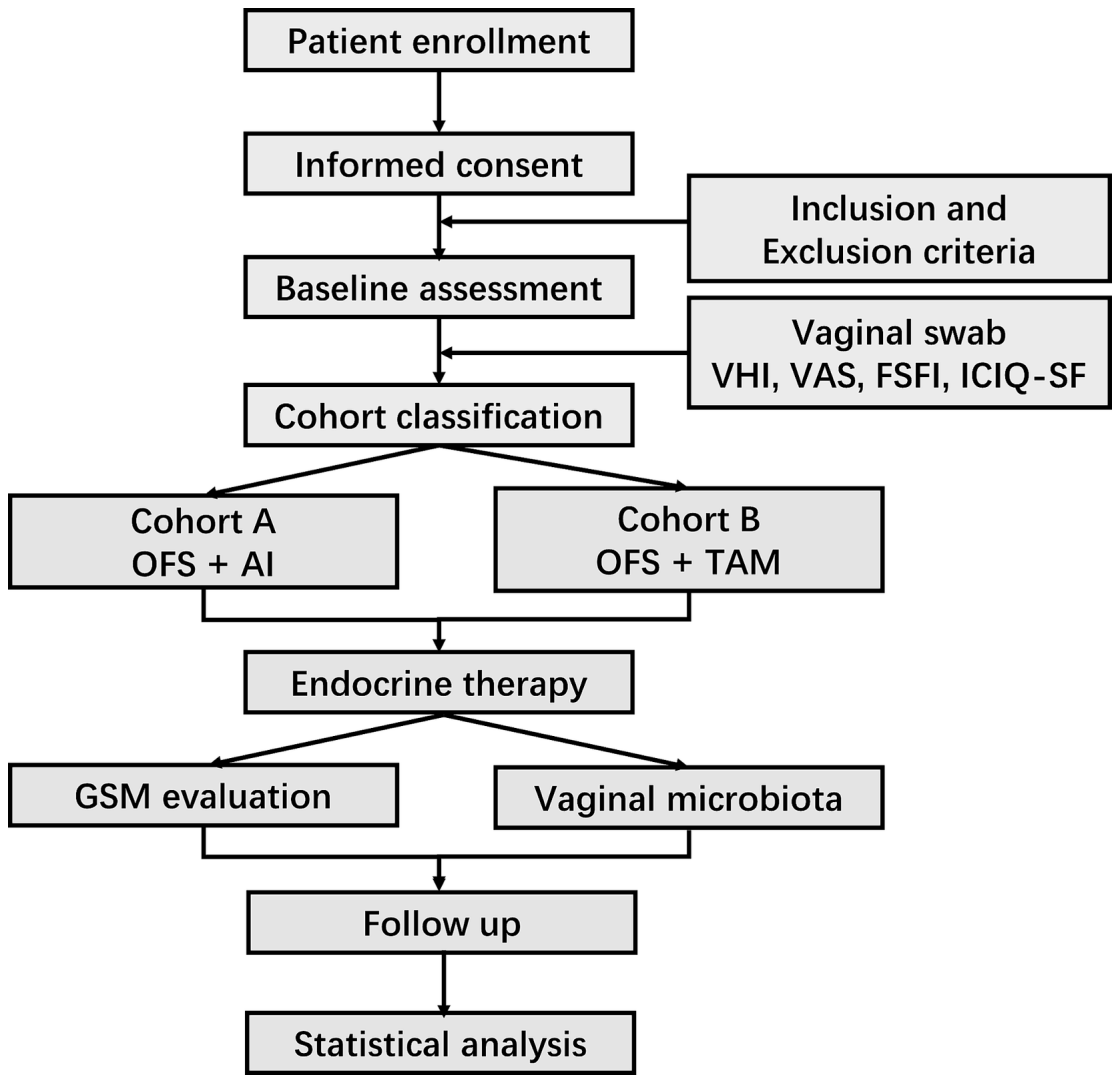
Flowchart of the study.

This study is a single-center, prospective, longitudinal cohort investigation aimed at establishing a cohort of premenopausal breast cancer patients under the age of 45, who are hormone receptor-positive and receiving either ovarian function suppression plus aromatase inhibitors or ovarian function suppression plus tamoxifen endocrine therapy (Figure 1). The research employs a prospective design, with patient recruitment set to begin on January 1, 2026, to develop a comprehensive clinical database and biospecimen repository. This study is set to be conducted at the Women’s Hospital, Zhejiang University School of Medicine. Patient recruitment begin from January 2026 and will continue until the target sample size is achieved (anticipated 1 to 2 years). Each enrolled participant will be followed up for a full 12 months, with planned study visits and sample collections at time point of 1 month, 3 months, 6 months, and 12 months. We ensure every participant can complete the full 12-month follow-up schedule.

The Women’s Hospital at Zhejiang University School of Medicine is designated as the primary coordinating center, tasked with overseeing the comprehensive study design, implementation, quality control, and data management. The research initiative will be spearheaded by a Principal Investigator (PI) who possesses specialized expertise in breast cancer research and clinical management. The PI will be responsible for the daily operations of the study, including participant recruitment, data collection, and quality assurance. Consistent with standard oncology practice in China, all patients receive comprehensive care managed by a multidisciplinary team (MDT). Following MDT consensus, our core research team consist of specialists from various clinical disciplines integral to the management of breast cancer, such as: breast surgeons with proficiency in oncologic surgery and systemic therapy, pathologists skilled in breast cancer diagnosis and molecular characterization, diagnostic radiologists with specialization in breast imaging, and gynecologists equipped to address the unique hormonal considerations in premenopausal patients, and so on.

All participants were mandated to provide written informed consent, comprehensively outlining the study’s objectives, procedures, potential risks, and benefits, thereby ensuring participants’ full comprehension of the study content and voluntary participation. For ancillary studies involving the collection of biological samples and genetic testing, distinct informed consent forms were developed to further protect participants’ rights to information and autonomous decision-making. The study protocol received approval from the Research.

Ethics Board of Women’s Hospital, Zhejiang University School of Medicine (IRB-20250423-R) and was conducted in strict adherence to the Declaration of Helsinki and pertinent national regulations. An independent safety monitoring committee was established to ensure adequate protection of subject safety and legal rights. In addition, a research quality and data quality monitoring committee, jointly established by the hospital’s ethics committee, medical affairs department, research department, and data management center, reviews our raw data every quarter to ensure the smooth progress of this study.

This study has been officially registered in the Chinese Clinical Trial Registry under the registration number ChiCTR2500115283, thereby ensuring transparency and traceability of the research process. In parallel, a comprehensive quality assurance framework has been implemented, which includes standardized operating procedures, regular training for the research team, stringent data quality control measures, periodic monitoring and auditing, and a robust documentation management system. These organizational structures and management strategies are designed to ensure the scientific rigor, regulatory compliance, and reliability of the study.

### Patient involvement

The inclusion criteria for the study are as follows: (1) Female participants aged between 18 and 45 years; (2) Histopathological confirmation of invasive breast carcinoma; (3) Positive status for hormone receptors, specifically estrogen receptor (ER) and/or progesterone receptor (PR); (4) Premenopausal status at diagnosis, characterized by baseline follicle-stimulating hormone levels below 40 IU/L and estradiol levels exceeding 20 pg./mL; (5) Scheduled to undergo ovarian function suppression in conjunction with endocrine therapy; (6) Absence of GSM-related symptoms prior to the initiation of treatment; (7) Provision of written informed consent by the participants.

Exclusion criteria included: (1) the presence of concurrent malignancies; (2) severe dysfunction of cardiac, hepatic, or renal systems; (3) pregnancy or lactation; (4) a history of vaginal infection; (5) the use of antibiotics or hormonal medications within the preceding 3 months; (6) an expected survival of less than 2 years [determined by the PREDICT tool^1^ or multidisciplinary team consensus]; (7) patients with psychiatric disorders or those who have been on active treatment with medications known to significantly alter sexual function (e.g., SSRIs, SNRIs); and (8) Patients with active high-risk HPV infections or abnormal cervical cytology requiring intervention.

This study employed a systematic recruitment strategy at Women’s Hospital, Zhejiang University School of Medicine, leveraging its role as a regional center for breast cancer care. Research coordinators reviewed pathology reports daily to identify newly diagnosed patients, conducting preliminary screenings based on age and demographics to compile a list of potential participants. Recruitment was timed post-diagnosis disclosure but pre-treatment planning to enhance receptivity, avoiding the day of diagnosis to reduce emotional burden.

The structured process included: (1) Initial clinician-led introduction during routine visits, with interested patients referred to the research team; (2) Face-to-face detailed explanations of study objectives, procedures, risks, and benefits by coordinators or nurses; (3) A minimum 24-h consideration period without pressure; (4) Signing of informed consent after addressing queries; and (5) Immediate baseline data collection and examinations. This approach aimed to minimize bias and ensure ethical enrollment of 110 participants over 6 months.

### Sample size

This study utilized a sample size calculation formula designed for comparing means between two independent groups. Our preliminary pilot-study data indicated a Simpson index of 0.8 in the OFS + AI group and 0.1 in the OFS + TAM group, with an effect size of *δ* = 0.7. Based on the literature-reported standard deviation of vaginal microbiota diversity (*σ* = 0.2), the effect size was calculated as d = δ/σ. The significance level was set at *α* = 0.05, with a statistical power of 1 - *β* = 0.8. The calculations suggested that 25 participants per group were necessary to detect a moderate effect size difference. Based on an annual cohort of 500 breast cancer patients, 20–30% (100–150 patients per year) are younger than 50 years. Over our planned 2-year recruitment period, we expect 200–300 eligible patients. Assuming a conservative recruitment rate of 40–50%, this allows us to successfully meet our target sample size of 110 participants (55 participants per group).

### Cohort classification

This study employed an observational prospective cohort design with non-randomized grouping based on real-world clinical practice. Patients were categorized into two groups according to the endocrine therapy regimen prescribed by their treating clinicians, taking into account individual disease characteristics, age, prior treatment history, and personalized clinical needs. The prescription of OFS + AI versus OFS + TAM is determined by the treating oncologist in accordance NCCN (3) based on clinicopathological risk factors. In brief: (1) OFS + AI is preferred for patients at high risk of recurrenceor those with contraindications to tamoxifen; (2) OFS + TAM is considered for patients with intermediate risk or those who cannot tolerate AI; (3) Patient preference and comorbidities (e.g., baseline cardiovascular risk, endometrial abnormalities) are taken into account. Any patient using oral contraceptives or levonorgestrel-releasing IUDs is required to discontinue/remove them prior to enrollment, since hormone receptor-positive breast cancer is a contraindication for systemic or local hormonal contraceptives.

To assess and address potential confounding, comprehensive baseline data were collected, including demographic characteristics (age, BMI, educational level, marital status), clinicopathological features (tumor size, lymph node status, TNM stage, histological grade), immunohistochemical markers (ER, PR, HER2 expression status, Ki-67 index), treatment-related factors (surgical approach, chemotherapy history, radiotherapy history), and laboratory parameters (estrogen levels). Patients who received (neo) adjuvant chemotherapy are eligible for inclusion. By strictly enforcing the hormonal inclusion criteria (FSH < 40 IU/L and Estradiol >20 pg./mL), we ensure that even patients with prior chemotherapy exposure are in a biochemically confirmed premenopausal state at baseline. For these patients, baseline sampling and assessment are performed at least 4 weeks after the last chemotherapy dose and prior to the initiation of endocrinotherapy. Chemotherapy history (regimen, number of cycles, dates) is recorded as a potential confounder, and subgroup analyses stratified by chemotherapy exposure will be performed.

A propensity score for receiving OFS + AI versus OFS + TAM will be estimated using a multivariable logistic regression model incorporating key baseline covariates, including age, BMI, tumor stage, prior chemotherapy exposure, and baseline ovarian function. Inverse Probability of Treatment Weighting (IPTW) will be used as the primary approach to balance baseline characteristics between the two groups. Stabilized weights will be calculated, which are truncated at the 1st and 99th percentiles to mitigate the influence of extreme weights. Standardized mean differences (SMD) will be assessed to confirm covariate balance, with an SMD < 0.1 considered acceptable.

### Data collection and management

Data were collected using a secure electronic case report form (eCRF) system built on a validated platform compliant with regulatory standards. All study personnel were trained in Good Clinical Practice and received specific instruction on eCRF completion prior to study initiation. Data entry was performed in real-time or shortly after each study visit by trained research coordinators. Built-in range checks, logical validations, and mandatory field requirements were implemented to minimize entry errors at the point of data capture.

Discrepancies and missing data triggered automated queries, which were resolved by site staff with reference to source documents. All data modifications were tracked via an audit trail that recorded the user, date, time, original value, and revised value. Access to the eCRF was role-based, with modification rights restricted to designated personnel; investigators had read-only access to their own site data after database lock.

Data quality was monitored through a multi-layered approach. Monthly central audits assessed completeness and protocol compliance. Source data verification was conducted on 10% of randomly selected participants, with a pre-specified acceptability threshold of >95% consistency between eCRF and source documents. Standard operating procedures governed data cleaning, query resolution, coding of adverse events (if applicable), and database lock. The research quality and data quality monitoring committee will review our raw data regularly.

Electronic data were stored on secure servers with restricted access, daily backups, and encryption both at rest and in transit. Physical and digital confidentiality measures were enforced in accordance with institutional ethics committee requirements and national data protection regulations. All study data will be retained for at least 10 years after study completion, after which they will be anonymized or destroyed per regulatory guidelines.

### Vaginal microbiome sample collection and storage

Participants were instructed to abstain from sexual intercourse, vaginal medication, and douching for 48 h prior to sampling. For patients who are menstruating at baseline, sampling was scheduled 3–7 days after the cessation of menstruation to minimize interference from the menstrual cycle. At follow-up visits, most participants will be amenorrheic due to OFS; however, if a participant reports any vaginal bleeding, sampling will be rescheduled until bleeding has ceased for at least 3 days. During the procedure, patients were placed in the lithotomy position. A sterile vaginal speculum was used to fully expose the cervix. A sterile cotton swab was rotated in the posterior vaginal fornix for 10 s to collect the specimen, since this anatomical reservoir pools cervicovaginal secretions, providing a highly representative and stable sample of the vaginal microbiome and mucosal microenvironment. The swab was immediately placed into a sterile tube containing DNA/RNA Shield™ preservative, temporarily stored at 4 °C, and transferred to a – 80 °C ultra-low temperature freezer within 4 h for long-term storage.

### Microbial DNA extraction and 16S rRNA gene sequencing

Total microbial DNA was extracted from vaginal swab samples using a bacterial DNA extraction kit according to the manufacturer’s instructions. DNA purity and concentration were assessed using a NanoDrop One spectrophotometer. Using the qualified DNA as a template, the V3–V4 region of the 16S rRNA gene was amplified via PCR using universal primers 338F (5′-ACTCCTACGGGAGGCAGCA-3′) and 806R (5′-GTACTACVGGGTWTCTAAT-3′). A unique barcode was attached to the reverse primer for each sample.

PCR reactions were performed in a 50 μL volume containing 25 μL of 2 × Premix Taq, 2 μL each of 10 μM forward and reverse primers, 50 ng of DNA template, and nuclease-free water. The thermal cycling profile consisted of an initial denaturation at 95 °C for 3 min, followed by 30 cycles of denaturation at 95 °C for 30 s, annealing at 53 °C for 30 s, and extension at 72 °C for 30 s, with a final extension at 72 °C for 5 min. PCR product concentration was determined using NanoDrop. Samples with different barcodes were mixed in equimolar ratios. The mixture was further purified using agarose gel electrophoresis and analyzed with GeneTools software (v4.03.05.0; SynGene, United States); samples showing a bright main band at approximately 470 bp were selected for subsequent steps. Sequencing libraries were generated using the ALFA-SEQ DNA Library Prep Kit (DL1010). Library quality was assessed using a Qubit 2.0 Fluorometer and an Agilent 2,100 Bioanalyzer system. Paired-end 250 bp (PE250) sequencing was performed on the Illumina NovaSeq 6,000 platform.

### Sequence processing and amplicon sequence variant (ASV) analysis

Raw reads were processed using Fastp (v0.14.1) and Cutadapt to remove adapters, primers, short reads (<100 bp), and low-quality reads (defined as reads containing >10% unknown bases or >20% bases with a *Q*-value <20). Denoising and Amplicon Sequence Variant (ASV) inference were performed using the DADA2 pipeline (via QIIME 2). Briefly, forward and reverse reads were trimmed, filtered, denoised, merged, and chimeric sequences were removed using the DADA2 plugin, which is highly recommended for vaginal microbiome research to accurately differentiate closely related Lactobacillus species. ASVs were then clustered at 100% sequence identity. Taxonomic assignment of ASVs was performed using the q2-feature-classifier plugin with the SILVA database (v138). Representative sequences for each ASV were aligned against the SILVA database (v138) for taxonomic annotation.

### Diversity analysis and community structure assessment

Alpha diversity was evaluated by calculating the Shannon Index and Simpson index based on the ASV abundance matrix to assess species richness and evenness within samples. Differences between groups were analyzed using the Kruskal-Wallis test. Beta diversity was assessed by calculating Bray-Curtis distances and weighted/unweighted UniFrac distances between samples and visualized using Principal Coordinates Analysis (PCoA). Permutational multivariate analysis of variance (PERMANOVA), implemented via the adonis function in the R vegan package, was used to quantitatively evaluate the variation in microbial community structure explained by clinical grouping. To ensure robustness, ASV-level analyses were compared with genus-level aggregated data as a sensitivity analysis.

### Primary outcome

This study identified vaginal microbial diversity and the relative abundance of Lactobacillus as the primary outcome measures. High-throughput sequencing of the 16S rRNA V3-V4 region was utilized, and bioinformatics analysis was conducted using the DADA2 pipeline via QIIME 2 to generate ASVs, enabling single-nucleotide resolution for quantitative assessment of alterations in vaginal microecological diversity. Species-level identification, particularly for Lactobacillus species, will be performed based on ASV taxonomic assignment. Simultaneously, the relative abundance of the Lactobacillus genus within the total bacterial community was analyzed to evaluate vaginal microecological homeostasis. Measurement time points were established at baseline, and at 1 month, 3 months, 6 months, and 12 months post-treatment, thereby creating a comprehensive longitudinal observation trajectory.

The collection of vaginal microbial samples followed a strictly standardized procedure: participants were instructed to abstain from sexual intercourse, vaginal medications, and douching for 48 h prior to sampling. Additionally, individuals who were menstruating were scheduled for sampling within a 3- to 7-day window following the cessation of menstruation to minimize interference from the menstrual cycle. During the sampling process, participants were positioned in the lithotomy position, and the cervix was fully exposed using a sterile vaginal speculum. A sterile cotton swab was rotated in the posterior vaginal fornix for a duration of 10 s, subsequently placed into a sterile tube containing DNA/RNA Shield preservative, temporarily stored at 4 °C, and then transferred to a – 80 °C ultra-low temperature freezer for long-term storage within 4 hours.

### Secondary outcomes

Prior to inclusion (at baseline), all patients undergo a systematic evaluation of GSM symptoms using standardized questionnaires, and only patients who report no GSM symptoms are eligible for inclusion. The clinical functional indicators comprised the Vaginal Health Index (VHI), Visual Analog Scale (VAS) scores for GSM, and International Consultation on Incontinence Questionnaire (ICIQ) scores for urinary symptoms. The VHI evaluated five dimensions through gynecological examination: vaginal discharge, pH value, epithelial integrity, moisture, and elasticity, with a cumulative score ranging from 5 to 25 points. Two attending gynecologists from our core multidisciplinary research team, each with over 10 years of clinical experience specializing in gynecological oncology and reproductive endocrinology, independently assessed the scores after undergoing protocol-specific standardized training to minimize inter-observer variability. The scores were then averaged, with measurements taken at time points baseline, 3 months, 6 months, and 12 months. GSM symptoms were assessed using a 10-cm VAS to independently evaluate subjective symptoms such as vaginal dryness, dyspareunia, and burning sensation, with scores ranging from 0 to 10 (0 indicating no symptoms and 10 indicating the most severe symptoms), completed by patients at each time point from baseline to 12 months. Urinary symptoms were evaluated using the International Consultation on Incontinence Questionnaire-Short Form (ICIQ-SF), with scores ranging from 0 to 21 points, measured at baseline, 3 months, 6 months, and 12 months, to encompass the full spectrum of genitourinary syndrome manifestations. Patients independently completed symptom questionnaires under the on-site guidance of research assistants, and comprehensive documentation of medication adherence, lifestyle changes, and other relevant factors potentially influencing vaginal microecology was maintained at each follow-up visit. The results are systematically documented in the electronic Case Report Form for all participants.

Routine monitoring encompassed a comprehensive evaluation of complete blood count, hepatic and renal function, lipid profile, and blood glucose levels, with intensified surveillance during the initial treatment phase—specifically at baseline and 1 month—to identify adverse drug reactions. Subsequent assessments were conducted at six-month intervals. The evaluation of hormone levels was pivotal in assessing the efficacy of endocrine therapy, involving the measurement of serum estradiol, follicle-stimulating hormone, luteinizing hormone, and testosterone using standardized electrochemiluminescence immunoassays (ECLIA) at baseline, 3 months post-treatment, and every 6 months thereafter to monitor the extent and persistence of ovarian function suppression (Table 1). Exploratory analyses will examine correlations between post-OFS hormone levels and vaginal microbiome diversity as well as GSM symptom severity. The inclusion of inflammatory markers, such as C-reactive protein and interleukins, into the testing panel was considered based on advancements in research and preliminary findings.

**Table 1:** Schedule of data collection.

Assessment category	Specific indicators	Baseline	1 Month	3 Months	6 Months	12 Months
Basic information	Demographics, medical history, tumor characteristics	✓	–	–	–	–
Treatment information	Endocrine therapy regimen, dosage, compliance	✓	✓	✓	✓	✓
Clinical examination	Vital signs, general condition	✓	✓	✓	✓	✓
Gynecological examination	Gynecological physical examination	✓	–	✓	✓	✓
	Vaginal health index (VHI)	✓	–	✓	✓	✓
	Urinary symptoms (ICIQ score)	✓	–	✓	✓	✓
Primary endpoint	Vaginal secretion sample collection	✓	✓	✓	✓	✓
	Vaginal pH measurement	✓	–	✓	✓	✓
Blood tests	Complete blood count, liver and kidney function	✓	✓	–	✓	✓
	Hormone levels	✓	–	✓	–	✓
Questionnaire	GSM symptoms (VAS score)	✓	✓	✓	✓	✓
Safety	Adverse event recording	–	✓	✓	✓	✓

### Statistical analysis

This study utilized both the full analysis set (FAS) and the per-protocol set (PPS) for statistical evaluation. The FAS will include all enrolled participants who receive at least one dose of treatment and provide baseline data, analyzed according to their assigned group to minimize selection bias. The PPS will comprise participants who complete the study per protocol, excluding those with major violations such as non-adherence to treatment or loss to follow-up exceeding predefined thresholds. All tests will be two-sided, with a significance level of *α* = 0.05. Descriptive statistics will summarize continuous variables as mean ± standard deviation for normally distributed data or as median (interquartile range) for skewed distributions. Categorical variables will be presented as frequencies and percentages.

Given the observational nature of this cohort study, baseline imbalances between groups will be assessed using standardized mean differences. If substantial imbalances are detected (standardized mean difference >0.1 for key covariates), propensity score matching will be applied to create balanced subgroups for comparative analyses, with propensity scores estimated via logistic regression incorporating variables such as age, BMI, tumor stage, hormone receptor status, and chemotherapy history.

Between-group comparisons will use independent samples t-tests or Mann–Whitney U tests for continuous variables, depending on data distribution and variance homogeneity. Chi-square or Fisher’s exact tests will be applied for categorical variables.

For longitudinal analyses, we plan to use linear mixed-effects models (LMM) for continuous clinical outcomes (e.g., GSM symptom scores) and generalized linear mixed models (GLMM) for microbiome features (e.g., relative abundance of key taxa). Therapy group, time point, and their interaction will be included as fixed effects to evaluate whether temporal trajectories differ by treatment type. Repeated measures ANOVA (RM-ANOVA) will be retained only as a secondary sensitivity analysis for complete-case datasets. If the proportion of missing data for a given outcome exceeds 10%, multiple imputation by chained equations (MICE) will be performed under the missing-at-random (MAR) assumption, with 20 imputed datasets and pooled estimates using Rubin’s rules.

Multivariable analyses will incorporate multiple linear regression and mixed-effects models, adjusting for confounders such as age, body mass index (BMI), tumor stage, hormone receptor status, and chemotherapy history. Stepwise regression will select variables to evaluate the independent effects of endocrine therapy regimens on vaginal microbiota changes. To address multiple comparisons across primary and secondary outcomes, Bonferroni correction will be applied where appropriate. Pre-specified subgroup analyses will be conducted, stratified by factors such as chemotherapy history and baseline estrogen levels, to explore heterogeneity of effects. Sensitivity analyses will verify result robustness, and subgroup findings will be interpreted cautiously as exploratory. All statistical analyses were executed using SPSS version 26.0 and R version 4.0 software.

## Discussion

This study protocol aims to address the knowledge gap in the existing literature regarding vaginal microbiota changes under pharmacologically induced hypoestrogenism. Most prior studies have focused on natural menopause populations and their associations between vaginal microbiota and GSM symptoms, such as cross-sectional analyses showing increased microbial diversity and significant Lactobacillus reduction in postmenopausal women, correlated with GSM (14, 15), or multicenter analyses confirming that non-Lactobacillus-dominant states increase GSM risk (16). However, longitudinal studies on endocrine therapy-induced “chemical menopause” in breast cancer patients are scarce; limited existing research, such as a 2024 cross-sectional analysis of postmenopausal breast cancer survivors showing increased vaginal abundance of Sneathia and Gardnerella in symptomatic patients, is constrained by small sample size and inability to infer causality (21); another 2021 Kazakhstan study observed Lactobacillus depletion in breast cancer patients but did not investigate relations to treatment or GSM (22). Through a prospective cohort design, this protocol adds to the emerging literature by systematically investigating the dynamic vaginal microbiota changes in premenopausal hormone receptor-positive breast cancer patients receiving OFS + AI or OFS + TAM therapy. Our study uniquely incorporates a direct comparison between OFS + AI and OFS + TAM regimens, a detailed dose–response analysis of microbiome shifts with GSM severity, and a comprehensive assessment of clinical functional outcomes including VHI, VAS, and urinary symptoms. We hypothesize that OFS + AI therapy will lead to more significant Lactobacillus reduction, increased microbial diversity, and exacerbated GSM symptoms, while OFS + TAM may offer protective effects due to its partial estrogenic activity (5, 8), thereby providing a scientific basis for personalized preventive strategies.

The potential impacts and translational value of this study protocol are evident in multiple clinical and scientific areas. First, by integrating 16S rRNA sequencing data with clinical parameters (such as VHI scores, GSM symptom VAS, and FSFI assessments), the protocol design supports the development of a microbiota-GSM prediction model for early risk stratification and personalized preventive interventions; for instance, longitudinal microbiota sampling (at baseline and 1, 3, 6, 12 months) can identify high-risk patients and guide targeted probiotic supplementation, thereby reducing treatment discontinuation rates from 30 to 15% (25). Second, the protocol provides microbiological evidence to optimize endocrine therapy selection, particularly for patients at elevated GSM risk, by comparing microbial dynamic changes (e.g., Lactobacillus relative abundance and diversity metrics) between the OFS + AI and OFS + TAM groups, facilitating personalized clinical decision-making. Third, the protocol’s standardized assessment framework (including repeated measures analysis of variance and generalized estimating equations) will establish vaginal microecology evaluation standards for Chinese premenopausal breast cancer patients, addressing the data gap in Asian populations and contributing longitudinal evidence to international guideline development.

This study boasts several strengths, primarily its prospective cohort design, which effectively captures the real-time effects of endocrine therapy on the dynamics of the vaginal microbiome. Additionally, with standardized assessment tools and a multidisciplinary team approach, we ensure the reliability and comprehensiveness of the data. The sample size and follow-up timeframes have also been carefully considered to adequately reflect changes in the vaginal microbiome and their associations with genitourinary syndrome of menopause (GSM) symptoms. This comprehensive approach provides valuable longitudinal evidence for both domestic and international guideline development.

Nonetheless, the study does have some limitations. Being a single-center investigation, its results may lack generalizability; thus, multicenter studies are needed to validate the findings. Furthermore, the 12-month follow-up period may not be sufficient to fully capture the long-term dynamics of the vaginal microbiome and GSM symptomatology. Additionally, the use of 16S rRNA gene sequencing is limited to the genus level, potentially overlooking critical species-level changes. Future research should incorporate multi-omics approaches, including metagenomics and metabolomics, to yield a more comprehensive analysis. To address these limitations, this study will apply statistical adjustments and sensitivity analyses to verify the robustness of the results.

Building upon the anticipated outcomes of this study, future research directions can be delineated into four principal areas. The first area involves the execution of multicenter randomized controlled trials aimed at validating the preventive efficacy of microbiota-targeted interventions. These interventions may include supplementation with specific Lactobacillus strains, personalized probiotic therapies, or the clinical application of microecological modulators. Such trials are essential to generate high-quality evidence that can inform evidence-based medical practice.

Secondly, the integration of multi-omics analytical technologies, including metagenomics, transcriptomics, and metabolomics, is essential for a comprehensive elucidation of the molecular mechanisms underlying microbiota-host interactions. This approach aims to clarify the pathophysiological pathways through which vaginal microecological dysbiosis contributes to genitourinary syndrome of menopause (GSM) symptoms, thereby establishing a foundation for the development of novel therapeutic targets.

Third, it is imperative to expand to international multicenter cohort studies that incorporate diverse racial and geographic populations. This approach will enhance the generalizability of research findings and inform the development of international guidelines. Simultaneously, it is crucial to explore the influence of genetic polymorphisms on vaginal microecology to advance precision medicine applications in the management of genitourinary syndrome of menopause (GSM).

Fourth, the development of digital health tools, including portable detection devices, artificial intelligence-based predictive algorithms, and telemedicine platforms, facilitates dynamic monitoring and personalized management of vaginal microecology. These advancements enhance convenience and adherence in the management of patient symptoms.

## Footnotes

1
www.predict.nhs.uk
